# Supplementary material for: In situ and in vitro cryo-EM reveal structures of mycobacterial encapsulin assembly intermediates
Source: Commun Biol. 2025 Feb 15;8:245. doi: 10.1038/s42003-025-07660-5 (PMC11830004; doi:10.1038/s42003-025-07660-5)
Supplement: Supplementary file 2 — Supplementary information [file 42003_2025_7660_MOESM2_ESM.pdf]

In situ and in vitro cryo-EM reveal structures of mycobacterial encapsulin assembly intermediates

Casper Berger[#1,3*], Chris Lewis[1,2*], Ye Gao[1], Kèvin Knoops[1,2], Carmen López-Iglesias[1,2], Peter J. Peters[1], Raimond B. G. Ravelli[1,4]

[1] Division of Nanoscopy, Maastricht Multimodal Molecular Imaging Institute, Maastricht University, Maastricht, The Netherlands
[2] Microscopy CORE Lab, FHML, Maastricht University, Maastricht, The Netherlands
[3] Current address: Structural Biology, The Rosalind Franklin Institute, Harwell Science & Innovation Campus, Didcot, United Kingdom
[4] Deceased: Raimond Ravelli

[*] These authors contributed equally
[#] To whom correspondence should be addressed: casper.berger@rfi.ac.uk

Supplementary Information

Supplementary Movies

Supplementary Movie 1-3. Structure of the 48-mer (Movie 1) 52-Mer (Movie 2) and the 54-mer (Movie3) with the residues coloured according to the RMSD value compared to the structure of the full shell (values in Å). Scalebar: 10 nm.
Supplementary Movie 4. Model of full encapsuling shell of *M. tuberculosis* and the intermediate structure of the 54-mer, showing that the 54-mer is slightly elongated in one direction, and shortened perpendicular to this direction in relation to the full encapsuling shell. Scalebar: 25 Å.

Supplementary Figures

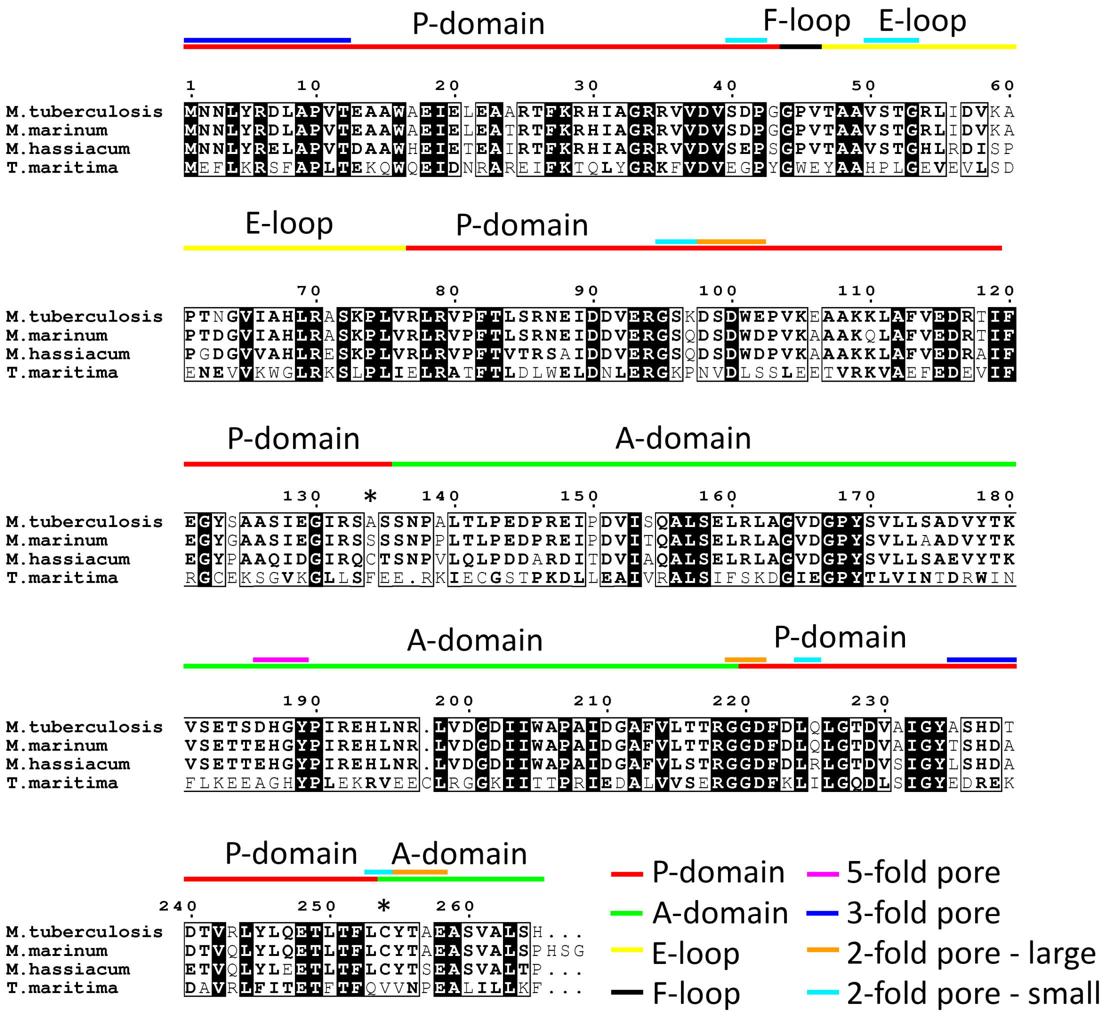

Supplementary Figure 1. Sequence alignment of Enc of different bacterial species. Identical sequences are shown in white and conserved sequences are shown in black with a black border. Variable and non-conserved amino acids are displayed in grey. The P-domain, A-domain, E-loop and F-loop are displayed as coloured lines above the sequence alignment. Regions lining the different pores are displayed as coloured lines right above the lines that indicate the different domains. Amino acids 134 and 254, which form a disulphide bridge in *M. hassiacum*, are denoted with a star.

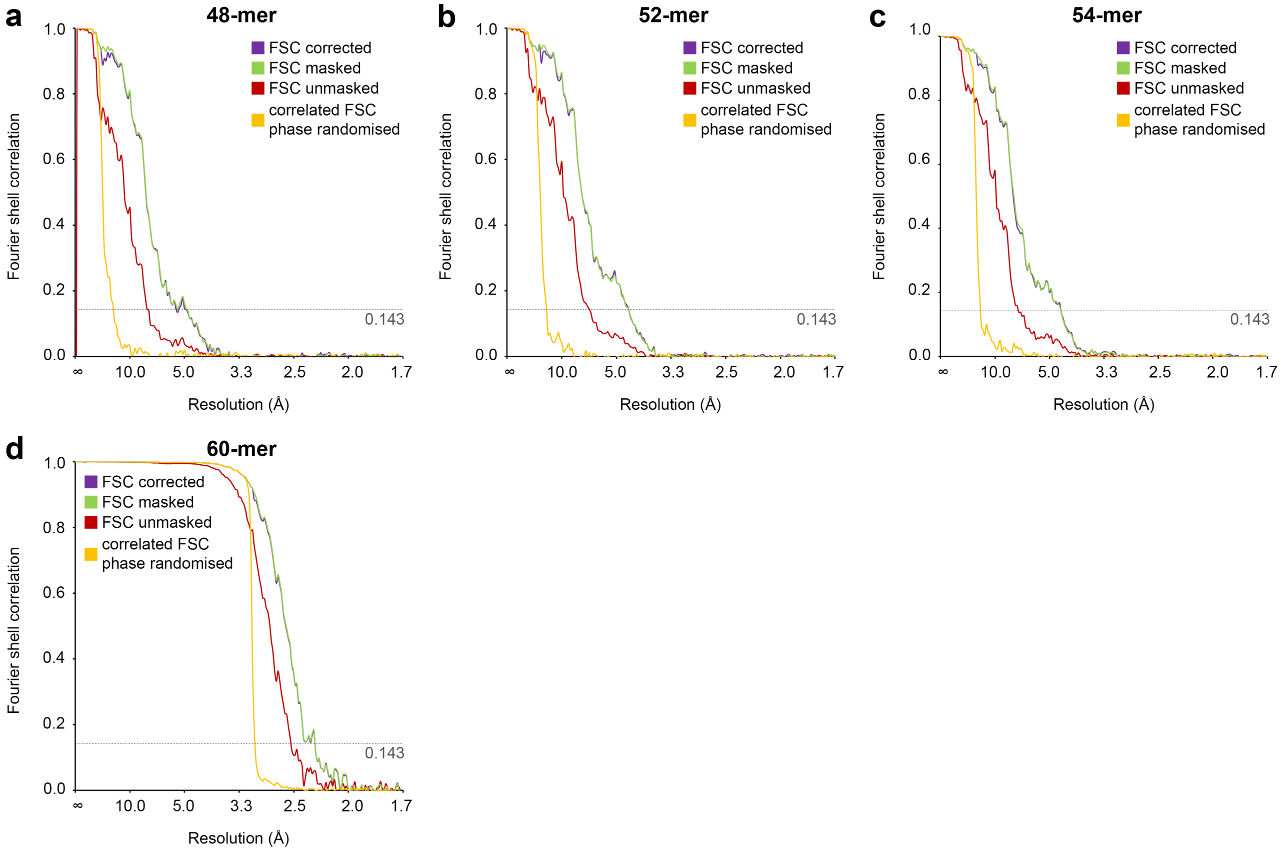

Supplementary Figure 2. Resolution of encapsulin shell cryo-EM single particle structures. Fourier shell correlations of the (a) 48-mer (5.4 Å), (b) 52-mer (4.5 Å), (c) 54-mer (4.6 Å), and (d) the full encapsulin shell (2.3 Å).

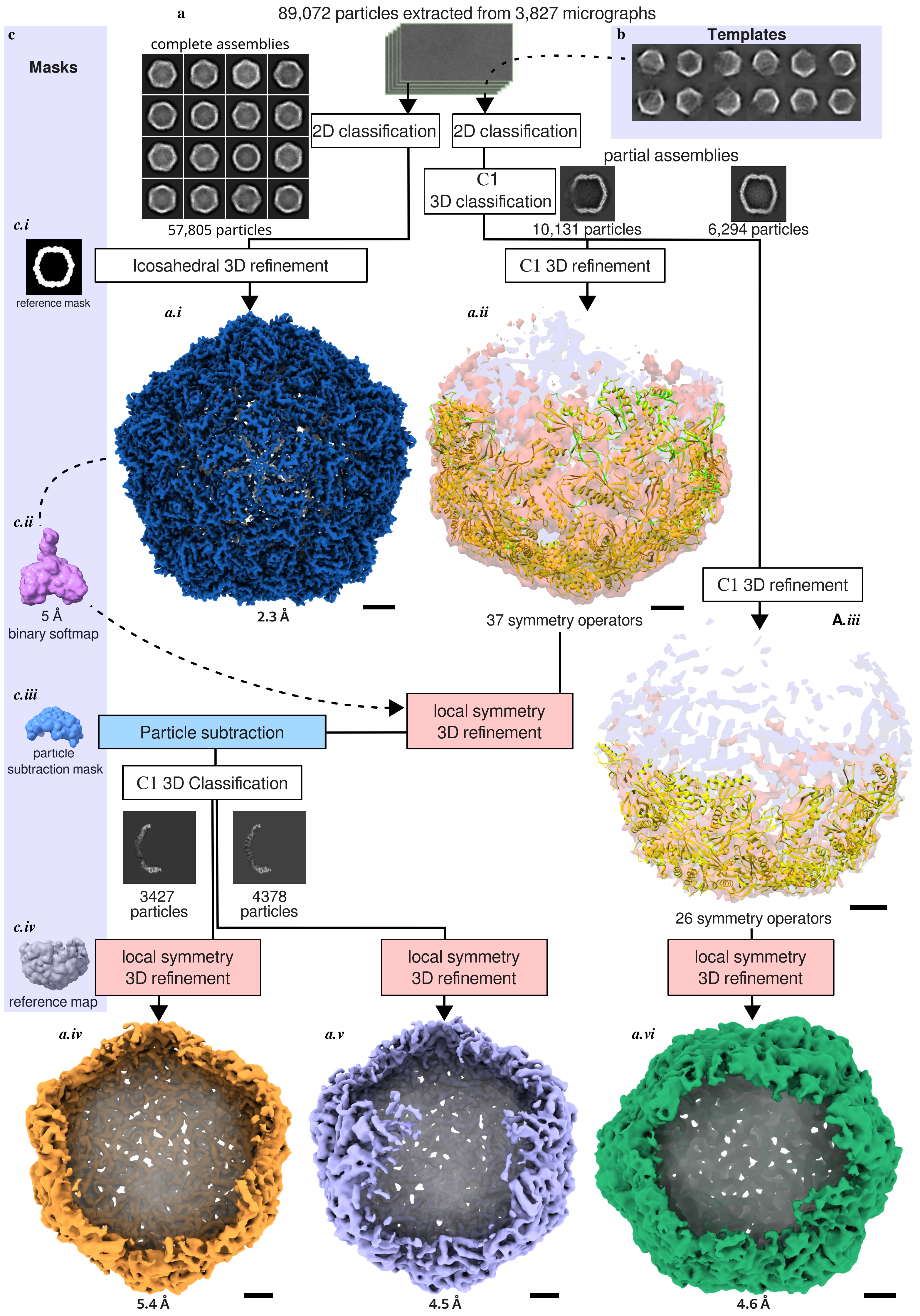

a
89,072 particles extracted from 3,827 micrographs
b
Templates
c
Masks
complete assemblies
57,805 particles
2D classification
2D classification
partial assemblies
C1
3D classification
10,131 particles
6,294 particles
c.i
reference mask
Icosahedral 3D refinement
C1 3D refinement
a.i
a.ii
c.ii
5 Å
binary softmap
2.3 Å
C1 3D refinement
37 symmetry operators
A.iii
c.iii
particle
subtraction mask
Particle subtraction
local symmetry
3D refinement
C1 3D Classification
3427
particles
4378
particles
26 symmetry operators
c.iv
reference map
local symmetry
3D refinement
local symmetry
3D refinement
local symmetry
3D refinement
a.iv
a.v
a.vi
5.4 Å
4.5 Å
4.6 Å

Supplementary Figure 3. Cryo-EM single particle analysis of the encapsulin shell from *M. tuberculosis.* (a) Flow chart of the methodology used to determine full and partial encapsulin shell assembly cryo-em maps, showing the dataset split into fully assembled and partially assembled encapsulin particles. (b) 12 Templates used for autopicking partial encapsulin assemblies. The templates were created by removing monomers from a model of the complete shell and creating 12 2D projections. (c) Masks were applied at their respective processing steps. Partially assembled particles were processed without masks and symmetry unless presented.

(a.i) The full encapsulin shell map (2.3 Å) was 3D refined from two 3D classes containing 34,427 particles with icosahedral symmetry applied. The dashed line shows production of a 5 Å binary softmap from our full encapsulin shell model, which was used to apply local symmetry during refinement of all partial encapsulin assemblies. (a.ii, a.iii) To ascertain where to apply symmetry, C1 refined maps (blue) from 3D classes were inspected against local occupancy maps (red). The yellow monomer chains fitted to the C1 maps denote where local symmetry operators will be applied in future 3D refinements. Minimal sets of symmetry operators were populated to apply symmetry away from the edges of the shell in regions of high occupancy. (a.iv and a.v) 5.4 Å and 4.5 Å maps of partial encapsulin assemblies with 37 symmetry operators applied during 3D refinement. For each particle the projection of its symmetrised region was subtracted from its experimental particle, and the result used for comparison during 3D classification focused on the non-symmetrised region. (a.vi) 4.6 Å map of a partial encapsulin assembly with 26 symmetry operators applied during 3D refinement. (c.i) The reference mask used for icosahedral 3D refinement of the complete encapsulin shell (c.ii) The binary softmap created from the Enc_{tb} monomer used in the application of local symmetry operators during 3D refinement of partially assembled encapsulin shells. (c.iii) The particle subtraction (white) mask used for focused 3D classification. (c.iv) The reference mask used for 3D classification of the subtracted particles. Scale bars: 25 Å.

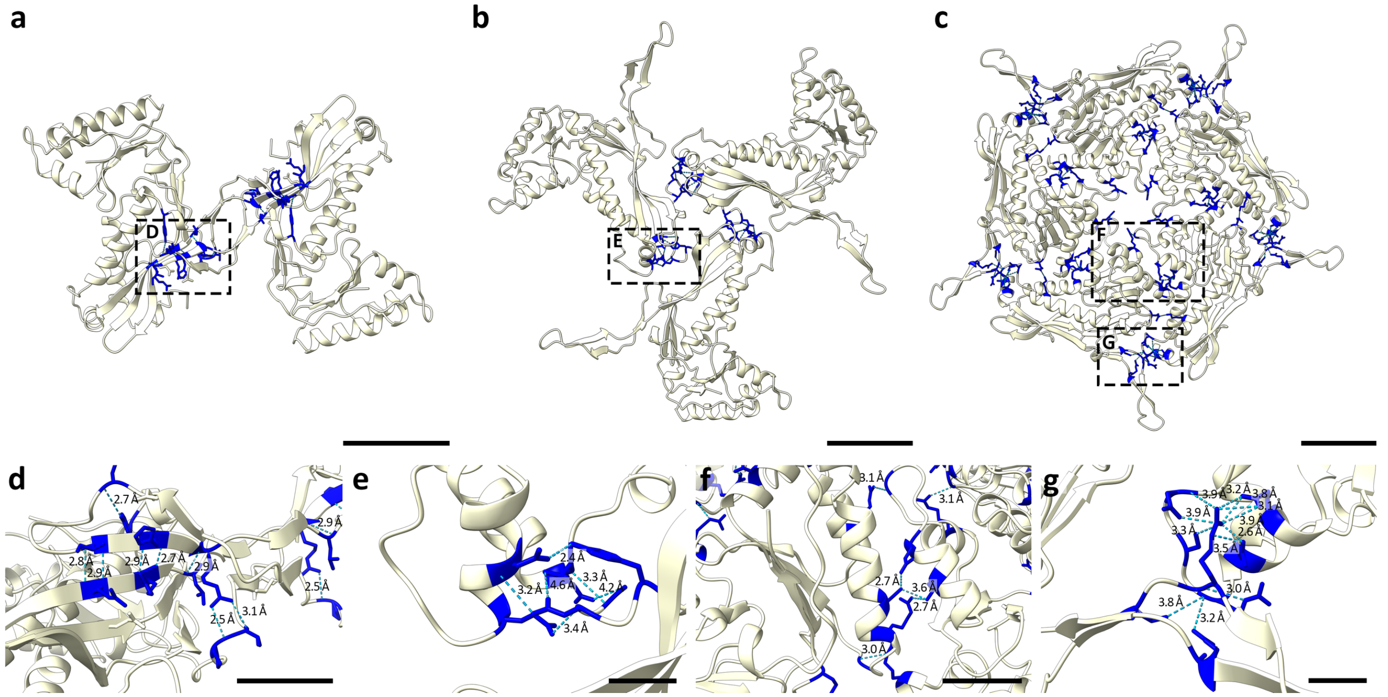

Supplementary Figure 4. Structure of Enc_{tb} binding interfaces with predicted bond formations. Structure of Enc_{tb} in dimeric (a), trimeric (b) and pentameric (c) conformation, with residues predicted to form intra-molecular hydrogen bonds by the PDBePISA server coloured in blue. Predicted hydrogen bonds are displayed as dashed lines in teal, with the bond distance shown in Å. Area's in Rectangles with black dashed lines in a-c are shown enlarged in panel d-g. There are 15 predicted intramolecular hydrogen bonds in the dimer interface via the E-loops but only 5 for the trimer. Although there are 18 predicted intramolecular hydrogen bonds for the pentameric interface, of which 8 are salt bridges, the distances for the hydrogen bonds are generally larger and the predicted solvation energy difference is smaller compared to the dimer interface. Scalebars: a-c: 25 Å, d and f: 10 Å, e and g: 5 Å.

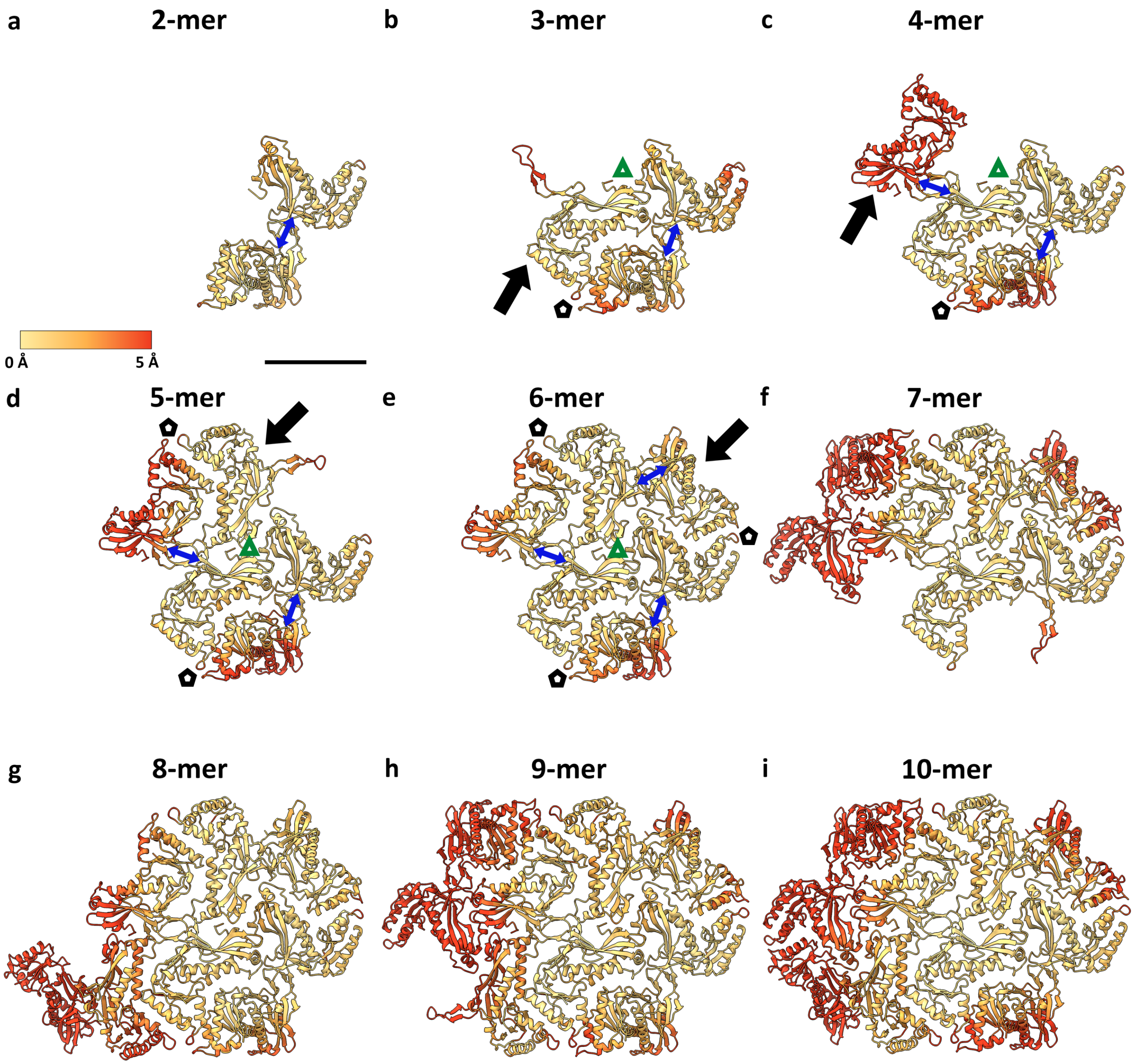

Supplementary Figure 5. Predicted multimeric Enc_{tb} structures. Alphafold multimer prediction for 2 to 10 copies of Enc_{tb} (a-i). Points of 2-fold, 3-fold and 5-fold symmetry around which Enc subunits are predicted to interact are indicated with blue double arrows (2-fold), green triangles (3-fold) and black pentagons (5-fold) in a-e. The local RMSD of the predicted multimer structure compared to the experimental cryo-EM structure obtained in this study is indicated in colour. Scalebar: 50 Å. (a) two copies of Enc_{tb}, predicted to form a dimer via the dimeric interface, in close accordance with the experimental structure (RMSD 0.88 Å for all atomic pairs). (b) Three copies of Enc_{tb}, predicted to form a dimer in accordance with the experimental structure, with an additional Enc_{tb} subunit interacting (black arrow) with the dimer via a trimeric and pentameric interface. RMSD for all atomic pairs: 1.42 Å. (c) Four copies of Enc_{tb}, predicted to form two dimers, which interact via the same trimeric and pentameric interface as predicted for three copies (RMSD 3.55 Å for all atomic pairs). (d) Five copies of Enc_{tb}, predicted to form the same two dimers that interact via a pentameric and trimeric interface as four copies, but with the addition of one Enc_{tb} subunit (black arrow) via a trimeric and pentameric interface. RMSD for all atomic pairs: 2.39 Å. (e) Six copies of $Enc_{tb,}$ predicted to form three dimers assembled around a trimeric interface (RMSD 1.27 Å for all atomic pairs). F-I RMSD values for all atomic pairs of 7 to 10 mers: 3.39 Å, 2.55 Å, 2,32 Å and 3.32 Å.

Supplementary Table 1. RELION Local Symmetry operators for encapsulin 48-mer. All symmetry operations are relative to their corresponding mask. The masks are included in the EMDB depositions.

_rlnMaskName	_rlnAngleRot	_rlnAngleTilt	_rlnAnglePsi
cfp29_molmap_Ref252_AV_5A_0p859_mask01.mrc	0	180	180
cfp29_molmap_Ref252_AV_5A_0p859_mask01.mrc	-110.91	60	69.09
cfp29_molmap_Ref252_AV_5A_0p859_mask01.mrc	-148.28	108	31.72
cfp29_molmap_Ref252_AV_5A_0p859_mask01.mrc	148.28	108	-31.72
cfp29_molmap_Ref252_AV_5A_0p859_mask01.mrc	110.91	60	-69.09
cfp29_molmap_Ref252_AV_5A_0p859_mask01.mrc	-69.09	120	69.09
cfp29_molmap_Ref252_AV_5A_0p859_mask01.mrc	110.91	120	-110.91
cfp29_molmap_Ref252_AV_5A_0p859_mask01.mrc	69.09	120	-69.09
cfp29_molmap_Ref252_AV_5A_0p859_mask01.mrc	31.72	72	-31.72
cfp29_molmap_Ref252_AV_5A_0p859_mask01.mrc	148.28	72	-148.28
cfp29_molmap_Ref252_AV_5A_0p859_mask01.mrc	-31.72	72	31.72
cfp29_molmap_Ref252_AV_5A_0p859_mask01.mrc	-148.28	72	148.28
cfp29_molmap_Ref252_AV_5A_0p859_mask01.mrc	-110.91	120	110.91
cfp29_molmap_Ref252_AV_5A_0p859_mask01.mrc	-148.28	144	-31.72
cfp29_molmap_Ref252_AV_5A_0p859_mask01.mrc	180	90	-90
cfp29_molmap_Ref252_AV_5A_0p859_mask01.mrc	-148.28	36	-148.28
cfp29_molmap_Ref252_AV_5A_0p859_mask01.mrc	-69.09	60	110.91
cfp29_molmap_Ref252_AV_5A_0p859_mask01.mrc	69.09	60	-110.91
cfp29_molmap_Ref252_AV_5A_0p859_mask01.mrc	-90	90	0
cfp29_molmap_Ref252_AV_5A_0p859_mask01.mrc	-31.72	36	-31.72
cfp29_molmap_Ref252_AV_5A_0p859_mask01.mrc	148.28	36	148.28
cfp29_molmap_Ref252_AV_5A_0p859_mask01.mrc	180	90	90
cfp29_molmap_Ref252_AV_5A_0p859_mask01.mrc	148.28	144	31.72
cfp29_molmap_Ref252_AV_5A_0p859_mask01.mrc	31.72	36	31.72
cfp29_molmap_Ref252_AV_5A_0p859_mask01.mrc	-148.28	36	31.72
cfp29_molmap_Ref252_AV_5A_0p859_mask01.mrc	148.28	72	31.72
cfp29_molmap_Ref252_AV_5A_0p859_mask01.mrc	90	90	0
cfp29_molmap_Ref252_AV_5A_0p859_mask01.mrc	31.72	144	-31.72
cfp29_molmap_Ref252_AV_5A_0p859_mask01.mrc	-148.28	72	-31.72
cfp29_molmap_Ref252_AV_5A_0p859_mask01.mrc	148.28	36	-31.72
cfp29_molmap_Ref252_AV_5A_0p859_mask01.mrc	31.72	108	31.72
cfp29_molmap_Ref252_AV_5A_0p859_mask01.mrc	-31.72	108	-31.72
cfp29_molmap_Ref252_AV_5A_0p859_mask01.mrc	0	90	-90
cfp29_molmap_Ref252_AV_5A_0p859_mask01.mrc	31.72	36	-148.28
cfp29_molmap_Ref252_AV_5A_0p859_mask01.mrc	110.91	60	110.91
cfp29_molmap_Ref252_AV_5A_0p859_mask01.mrc	110.91	120	69.09
cfp29_molmap_Ref252_AV_5A_0p859_mask01.mrc	0	0	-180
cfp29_molmap_Ref252_AV_5A_0p859_mask01.mrc	69.09	60	69.09

Supplementary Table 2. RELION Local Symmetry operators for encapsulin 52-mer. All symmetry operations are relative to their corresponding mask. The masks are included in the EMDB depositions.

**_rlnMaskName**	**_rlnAngleRot**	**_rlnAngleTilt**	**_rlnAnglePsi**
cfp29_molmap_Ref253_AV_5A_0p859_mask01.mrc	0	180	180
cfp29_molmap_Ref253_AV_5A_0p859_mask01.mrc	-110.91	60	69.09
cfp29_molmap_Ref253_AV_5A_0p859_mask01.mrc	-148.28	108	31.72
cfp29_molmap_Ref253_AV_5A_0p859_mask01.mrc	148.28	108	-31.72
cfp29_molmap_Ref253_AV_5A_0p859_mask01.mrc	110.91	60	-69.09
cfp29_molmap_Ref253_AV_5A_0p859_mask01.mrc	-69.09	120	69.09
cfp29_molmap_Ref253_AV_5A_0p859_mask01.mrc	110.91	120	-110.91
cfp29_molmap_Ref253_AV_5A_0p859_mask01.mrc	69.09	120	-69.09
cfp29_molmap_Ref253_AV_5A_0p859_mask01.mrc	31.72	72	-31.72
cfp29_molmap_Ref253_AV_5A_0p859_mask01.mrc	148.28	72	-148.28
cfp29_molmap_Ref253_AV_5A_0p859_mask01.mrc	-31.72	72	31.72
cfp29_molmap_Ref253_AV_5A_0p859_mask01.mrc	-148.28	72	148.28
cfp29_molmap_Ref253_AV_5A_0p859_mask01.mrc	-110.91	120	110.91
cfp29_molmap_Ref253_AV_5A_0p859_mask01.mrc	-148.28	144	-31.72
cfp29_molmap_Ref253_AV_5A_0p859_mask01.mrc	180	90	-90
cfp29_molmap_Ref253_AV_5A_0p859_mask01.mrc	-148.28	36	-148.28
cfp29_molmap_Ref253_AV_5A_0p859_mask01.mrc	-69.09	60	110.91
cfp29_molmap_Ref253_AV_5A_0p859_mask01.mrc	69.09	60	-110.91
cfp29_molmap_Ref253_AV_5A_0p859_mask01.mrc	-90	90	0
cfp29_molmap_Ref253_AV_5A_0p859_mask01.mrc	-31.72	36	-31.72
cfp29_molmap_Ref253_AV_5A_0p859_mask01.mrc	148.28	36	148.28
cfp29_molmap_Ref253_AV_5A_0p859_mask01.mrc	180	90	90
cfp29_molmap_Ref253_AV_5A_0p859_mask01.mrc	148.28	144	31.72
cfp29_molmap_Ref253_AV_5A_0p859_mask01.mrc	31.72	36	31.72
cfp29_molmap_Ref253_AV_5A_0p859_mask01.mrc	-148.28	36	31.72
cfp29_molmap_Ref253_AV_5A_0p859_mask01.mrc	148.28	72	31.72
cfp29_molmap_Ref253_AV_5A_0p859_mask01.mrc	90	90	0
cfp29_molmap_Ref253_AV_5A_0p859_mask01.mrc	31.72	144	-31.72
cfp29_molmap_Ref253_AV_5A_0p859_mask01.mrc	-148.28	72	-31.72
cfp29_molmap_Ref253_AV_5A_0p859_mask01.mrc	148.28	36	-31.72
cfp29_molmap_Ref253_AV_5A_0p859_mask01.mrc	31.72	108	31.72
cfp29_molmap_Ref253_AV_5A_0p859_mask01.mrc	-31.72	108	-31.72
cfp29_molmap_Ref253_AV_5A_0p859_mask01.mrc	0	90	-90
cfp29_molmap_Ref253_AV_5A_0p859_mask01.mrc	31.72	36	-148.28
cfp29_molmap_Ref253_AV_5A_0p859_mask01.mrc	110.91	60	110.91
cfp29_molmap_Ref253_AV_5A_0p859_mask01.mrc	110.91	120	69.09
cfp29_molmap_Ref253_AV_5A_0p859_mask01.mrc	0	0	-180
cfp29_molmap_Ref253_AV_5A_0p859_mask01.mrc	69.09	60	69.09

Supplementary Table 3. RELION Local Symmetry operators for encapsulin 54-mer. All symmetry operations are relative to their corresponding mask. The masks are included in the EMDB depositions.

_rlnMaskName	_rlnAngleRot	_rlnAngleTilt	_rlnAnglePsi
cfp29_molmap_Ref168_flipHand_AV_5A_0p859_mask01.mrc	-110.91	60	69.09
cfp29_molmap_Ref168_flipHand_AV_5A_0p859_mask01.mrc	-148.28	108	31.72
cfp29_molmap_Ref168_flipHand_AV_5A_0p859_mask01.mrc	148.28	108	-31.72
cfp29_molmap_Ref168_flipHand_AV_5A_0p859_mask01.mrc	110.91	60	-69.09
cfp29_molmap_Ref168_flipHand_AV_5A_0p859_mask01.mrc	148.28	72	-148.28
cfp29_molmap_Ref168_flipHand_AV_5A_0p859_mask01.mrc	-31.72	72	31.72
cfp29_molmap_Ref168_flipHand_AV_5A_0p859_mask01.mrc	-148.28	72	148.28
cfp29_molmap_Ref168_flipHand_AV_5A_0p859_mask01.mrc	-110.91	120	110.91
cfp29_molmap_Ref168_flipHand_AV_5A_0p859_mask01.mrc	180	90	-90
cfp29_molmap_Ref168_flipHand_AV_5A_0p859_mask01.mrc	-148.28	36	-148.28
cfp29_molmap_Ref168_flipHand_AV_5A_0p859_mask01.mrc	-69.09	60	110.91
cfp29_molmap_Ref168_flipHand_AV_5A_0p859_mask01.mrc	69.09	60	-110.91
cfp29_molmap_Ref168_flipHand_AV_5A_0p859_mask01.mrc	-31.72	36	-31.72
cfp29_molmap_Ref168_flipHand_AV_5A_0p859_mask01.mrc	148.28	36	148.28
cfp29_molmap_Ref168_flipHand_AV_5A_0p859_mask01.mrc	180	90	90
cfp29_molmap_Ref168_flipHand_AV_5A_0p859_mask01.mrc	148.28	144	31.72
cfp29_molmap_Ref168_flipHand_AV_5A_0p859_mask01.mrc	31.72	36	31.72
cfp29_molmap_Ref168_flipHand_AV_5A_0p859_mask01.mrc	-148.28	36	31.72
cfp29_molmap_Ref168_flipHand_AV_5A_0p859_mask01.mrc	148.28	72	31.72
cfp29_molmap_Ref168_flipHand_AV_5A_0p859_mask01.mrc	90	90	0
cfp29_molmap_Ref168_flipHand_AV_5A_0p859_mask01.mrc	-148.28	72	-31.72
cfp29_molmap_Ref168_flipHand_AV_5A_0p859_mask01.mrc	148.28	36	-31.72
cfp29_molmap_Ref168_flipHand_AV_5A_0p859_mask01.mrc	110.91	60	110.91
cfp29_molmap_Ref168_flipHand_AV_5A_0p859_mask01.mrc	110.91	120	69.09
cfp29_molmap_Ref168_flipHand_AV_5A_0p859_mask01.mrc	0	0	-180
cfp29_molmap_Ref168_flipHand_AV_5A_0p859_mask01.mrc	69.09	60	69.09
