# Supplementary material for: In situ and in vitro cryo-EM reveal structures of mycobacterial encapsulin assembly intermediates
Source: Commun Biol. 2025 Feb 15;8:245. doi: 10.1038/s42003-025-07660-5 (PMC11830004; doi:10.1038/s42003-025-07660-5)
Supplement: Supplementary file 3 — Description of Additional Supplementary Files [file 42003_2025_7660_MOESM3_ESM.pdf]

Description of Additional Supplementary Files

File name: Supplementary Movies 1-3
Description: Structure of the 48-mer (Movie 1) 52-Mer (Movie 2) and the 54-mer (Movie3) with the residues coloured according to the RMSD value compared to the structure of the full shell (values in Å). Scalebar: 10 nm.

File name: Supplementary Movie 4
Description: Model of full encapsuling shell of *M. tuberculosis* and the intermediate structure of the 54-mer, showing that the 54-mer is slightly elongated in one direction, and shortened perpendicular to this direction in relation to the full encapsuling shell. Scalebar: 25 Å.
