# Supplementary figures and images for: In situ and in vitro cryo-EM reveal structures of mycobacterial encapsulin assembly intermediates
Source: Commun Biol. 2025 Feb 15;8:245. doi: 10.1038/s42003-025-07660-5 (PMC11830004; doi:10.1038/s42003-025-07660-5)

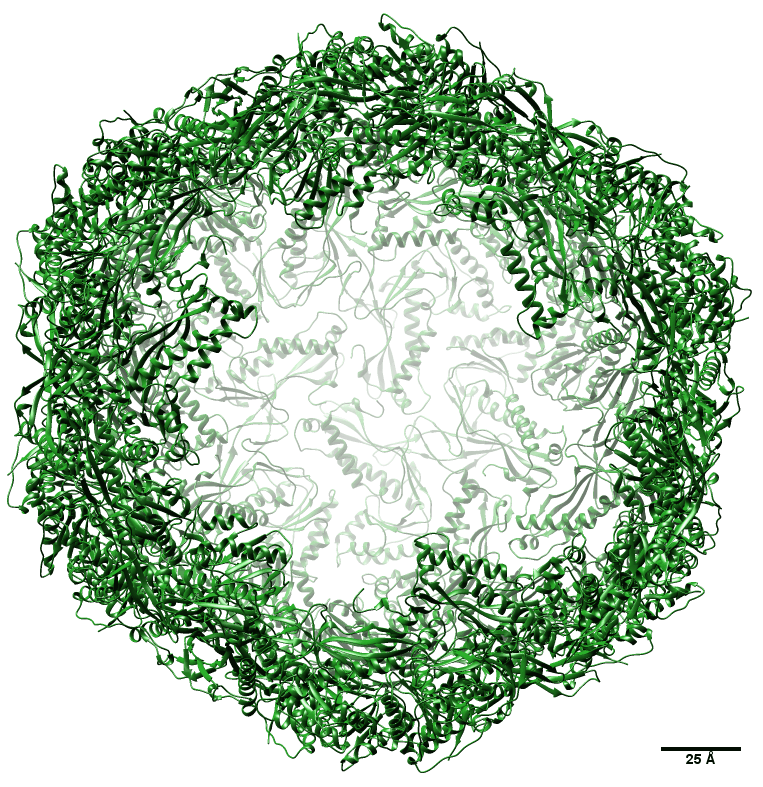

Supplement: Supplementary file 7 — Supplementary Movie 4 [file 42003_2025_7660_MOESM7_ESM.gif]
